# Two-year randomized controlled trial of circumferential versus segmental ab externo angle surgery in patients with primary open angle glaucoma

**DOI:** 10.1007/s10384-024-01150-7

**Published:** 2025-01-23

**Authors:** Ahmed Samy Elwehidy, Nader Hussein Lotfy Bayoumi, Mostafa A. S. Elwehidy, Nashaat Shawky Zaky, Sherein M. Hagras, Nader H. L. Bayoumi

**Affiliations:** 1 Faculty of Medicine, Mansoura University, Mansoura, Egypt; 2 Faculty of Medicine, Alexandria University, Alexandria, Egypt; 3 Resident of Ophthalmology, Faculty of Medicine, Mansoura University, Mansoura, Egypt

**Keywords:** Primary open angle glaucoma, Trabeculotomy, Viscotrabeculotomy, Intraocular pressure

## Abstract

**Purpose:**

To compare the surgical outcomes of visco-circumferential-suture-trabeculotomy (VCST) and rigid probe segmental viscotrabeculotomy (VT) in primary open-angle glaucoma (POAG).

**Study design:**

A prospective randomized controlled study.

**Patients and Methods:**

Patients presenting with POAG and operated upon in Mansoura Ophthalmic Center in Mansoura, Egypt between February 2017 and September 2021 were enrolled. Study eyes were randomized to either VCST or VT and follow up extended for 24 months. Success was defined as an intra ocular pressure (IOP)≤18 mmHg with a 40% reduction from baseline. Complications were noted.

**Results:**

The study enrolled 166 (82 in the circumferential group) eyes of 107 patients. There were no statistically significant differences between both groups in any demographic or preoperative clinical characteristics. The IOP trend demonstrated marked initial reduction (from 32.3±2.4 and 31.8±2.6 mmHg preoperatively in VCST and VT groups respectively) followed by a slow gradual rise over subsequent follow-up settling at lower IOP values in the circumferential (12.4±1.2 mmHg) than in the segmental group (15.5±0.9 mmHg) (p<0.001), both settling at significantly lower levels than preoperative values. The survival curve demonstrates complete success rates at 12, 15, 18, and 24 months were 98.8%, 96.4%, 95.1%, and 85.4% in the circumferential group and 98.8%, 92.9%, 91.7%, 79.8% in the segmental group respectively. Minimal self-limiting hyphema was universal in all study eyes.

**Conclusions:**

Angle procedures -segmental and circumferential- are effective in lowering the IOP for at least 2 years in eyes with POAG, with circumferential angle surgery providing a greater reduction of IOP and requiring fewer subsequent glaucoma procedures.

## Introduction

Glaucoma is the leading cause of irreversible visual loss worldwide. Primary open-angle glaucoma (POAG) is the most common type, accounting for 74% of all glaucoma cases [1, 2]. Trabeculectomy is still the most commonly performed glaucoma surgery worldwide [3]. However, it has several vision-threatening complications such as ocular hypotony, choroidal detachment, and bleb-related infections [4]. Therefore, in recent years, bleb-independent less invasive procedures, such as canaloplasty [5, 6], gonioscopy-assisted transluminal trabeculotomy (GATT) [7, 8], and ab externo circumferential trabeculotomy [6, 9, 10] have become increasingly common because of their greater efficacy and excellent safety profile. Despite successful outcomes and few postoperative complications of GATT in juvenile and adult POAG, surgeons with less experience performing GATT procedures may initially struggle with it. Additionally, GATT requires a reasonably clear cornea to visualize the nasal angle and introduce instruments into the anterior chamber (AC) which increases the risk of damage to intraocular structures [11, 12]. Over the years, many authors have demonstrated the efficacy of ab-externo angle procedures in lowering intraocular pressure (IOP) in congenital [13–15], juvenile [17], and adult open-angle glaucomas [6, 9, 10, 16]. Ab externo angle surgery includes both segmental and circumferential techniques as conventional rigid probe trabeculotomy or viscotrabeculotomy (VT) [13, 14, 17, 18], 360-degree suture trabeculotomy [9, 10], visco-circumferential-suture-trabeculotomy (VCST) [14, 17] and circumferential trabeculotomy with an illuminated microcatheter [15, 16, 19, 20]. Although the illuminated microcatheter helps to cannulate Schlemm’s canal (SC) safely, its cost constitutes a financial burden worldwide [15]. Visco-circumferential-suture-trabeculotomy (VCST) and circumferential trabeculotomy are proven to be effective in the reduction of IOP in both primary congenital glaucoma (PCG) and adult open-angle glaucoma as these procedures offer the advantages of performing 360^o^-circumferential trabeculotomy with great ease and at a low cost [9, 10, 14, 17].The aim of the present study was to compare the surgical outcomes of visco-circumferential-suture-trabeculotomy (VCST) and rigid probe segmental viscotrabeculotomy (VT) in patients with POAG.

## Patients and methods

This prospective randomized comparative study was conducted on 166 eyes of 107 patients with medically uncontrolled POAG diagnosed and operated upon in Mansoura Ophthalmic Center of Mansoura University, Egypt between February 2017 and September 2021. The study approval was obtained from the Ethical Committee of the Faculty of Medicine of Mansoura University according to the tenets of the Declaration of Helsinki. All patients in the study received a clear explanation of the study design, the surgical procedures, and their possible consequences and gave written informed consent.

All patients underwent a full ophthalmological examination, including the best corrected visual acuity (BCVA) using the decimal notation, slit lamp examination, intraocular pressure (IOP) measurement by Goldmann applanation tonometry (GAT), gonioscopy using Goldmann 3-mirrors goniolens for angle grading using Schaffer’s grading system and fundus examination. Visual field (VF) assessment was performed by SITA strategy perimetry (Humphrey, central 24 − 2 standard strategy). Peripapillary Retinal nerve fiber layer (pRNFL) thickness and optic disc were evaluated using a spectral domain optical coherence tomography (OCT; Topcon). The number of antiglaucoma medications (AGMs) was recorded.

Eyes with POAG, with a gonioscopic open angle, an IOP above 21 mm Hg despite the maximally tolerated AGM, typical glaucomatous VF defects, and glaucomatous optic disc appearance, in the absence of any obvious cause for glaucoma, were included in the study. Patients with primary angle closure glaucoma (PACG), secondary glaucomas, and who were on anticoagulant therapy and could not stop treatment or with media opacity that interfered with VF testing or OCT imaging were excluded from the study. Study eyes were randomized (by random number generator) to either VCST (circumferential group) or segmental VT (segmental).

In patients undergoing surgery in both eyes (59 patients), randomization to either VCST or VT was applied to the first operated eye while the other eye was assigned to the other procedure and included in the study. All surgical procedures were performed by the same experienced glaucoma surgeon (A.S.E.).

The criteria for complete success were defined as an intraocular pressure between 6 and 18 mmHg, with a reduction of at least 40% from the baseline IOP without the use of IOP-lowering medications or further surgery, and without any vision-threatening complications. Qualified success was defined as the same criteria for complete success but with the use of IOP-lowering medications. Additionally, success percentages were further defined as an IOP between 6 and 14 mmHg and with a reduction of at least 40% from baseline IOP. Failure was defined as failure to meet the success criteria on at least 2 consecutive adequately spaced visits, or as the occurrence of any vision-threatening complication (e.g. endophthalmitis, retinal detachment, etc.).

### Surgical techniques

Surgical techniques of both Circumferential-Suture-Viscotrabeculotomy (VCST) and rigid probe Viscotrabeculotomy (VT) were previously described in detail [13, 17, 18]. Supplemental Digital Content is a video showing excerpts from the surgery in one of the eyes of the current study. In eyes randomly chosen to undergo VCST where the procedure could not be completed, the procedure was converted to rigid probe VT -as a rescue- to achieve segmental ab externo angle surgery and the eye was excluded from the study (13 eyes). For eyes suffering a visually significant cataract the glaucoma procedure was combined with phacoemulsification (8 eyes (9.5%) in the VT group and 9 eyes (10.9%) in the VCST group). Postoperative treatment involved the application of topical ofloxacin and dexamethasone five times daily with gradual tapering over four weeks. Timolol and Dorzolamide drops were administered twice daily with or without latanoprost drops whenever required to control IOP spikes.

### Statistical analysis

Data were analyzed using SPSS software v.16.00. Quantitative variables were represented as the mean ± standard deviation (SD) while categorical variables were denoted by number and percentage. Kolmogorov–Smirnov tests were run to test the normal distribution of data. for normally distributed-variables; independent T-test was applied intergroup and independent-t test was used to describe the difference between the values at the end of the follow up and the baseline values within each group. Chi square test was applied to compare categorical variables. For all the tests used; *P* ≤ 0.0500 was considered statistically significant.

## Results

**Fig. 1:**
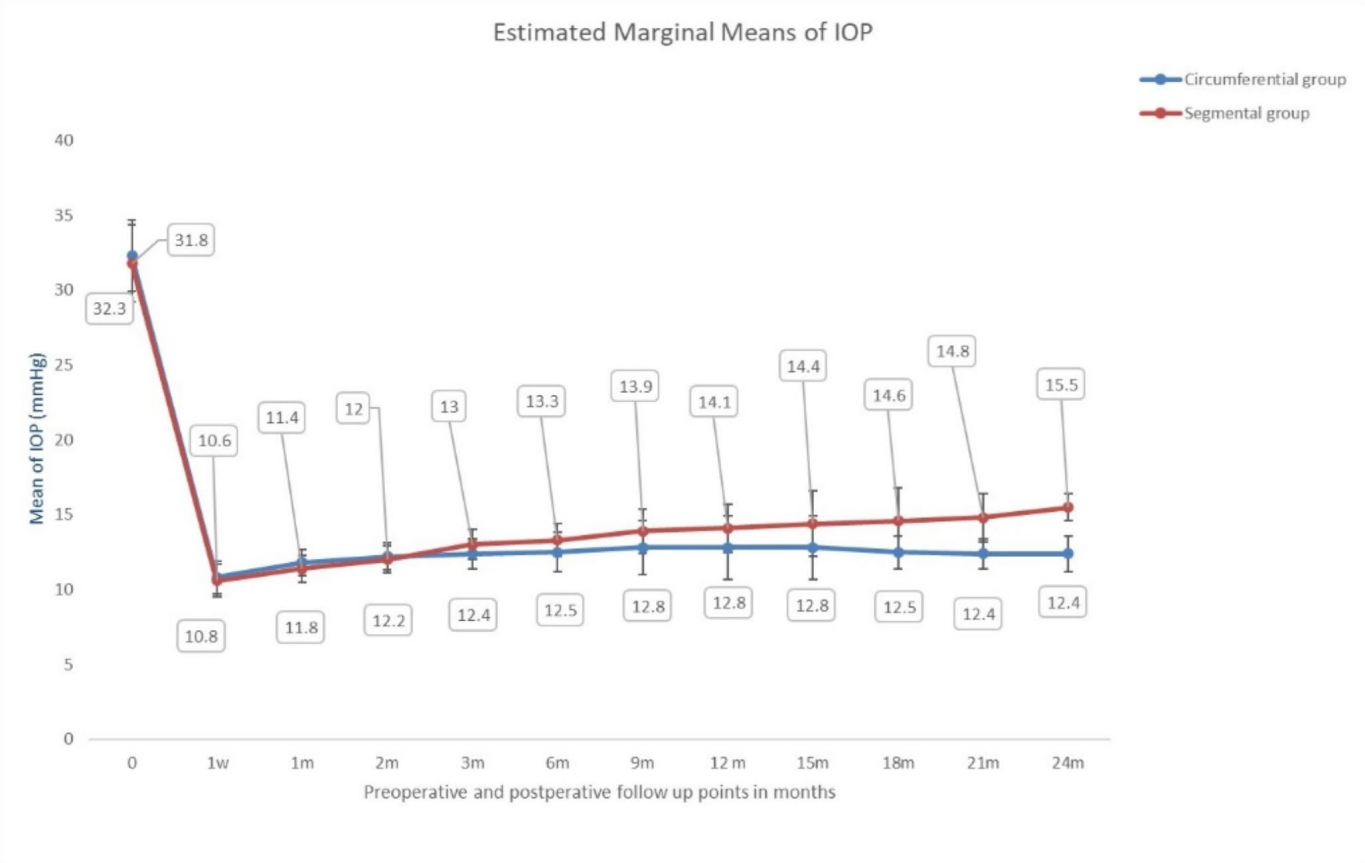
The distributions of the baseline tomographic and biomechanical parameters for both groups (Non-progressed and Progressed)

The study enrolled 166 (82 in the circumferential group) eyes of 107 (59 with both eyes enrolled in the study) patients presenting with and operated upon for POAG in Mansoura Ophthalmic Center in Mansoura, Egypt. The demographic and preoperative clinical characteristics of the study patients and eyes are presented in Table 1. There were no statistically significant differences between the circumferential group and the segmental group in any of the demographic or preoperative clinical characteristics. The postoperative IOP at the different follow up time points is presented in Table 2 (Fig. 1). The IOP trend demonstrates a marked initial reduction followed by a slow gradual rise over subsequent follow up time points settling at a lower IOP value in the circumferential than in the segmental group, both settling at a significantly lower level than the preoperative values. A statistically significant difference in postoperative IOP between the circumferential group and the segmental group starts at the 6th postoperative month onwards till the 24th month of follow up and till the final follow up time point. The number of AGMs at the end of the follow up in both groups was significantly less than the preoperative values (*p* < 0.001) whereas there was no statistically significant difference between the number of AGMs at the final follow up time point between the 2 study groups (*p* = 0346). At 24 months of follow up, the BCVA improved in both groups, however, the difference was not statistically significant (*p* > 0.05). There was no statistically significant difference between the 2 study groups in postoperative BCVA (*p* = 0.184). The VF global indices (Mean Deviation (MD) and Pattern Standard Deviation (PSD)) did not demonstrate a significant difference between the 2 study groups. However, the postoperative MD differed significantly from the preoperative values (*p* < 0.001) in the circumferential group, but not in the segmental group (*p* = 0.432) and the postoperative PSD improved significantly in both groups (*p* < 0.001 in both groups).

**Table 1:** Demographic and baseline (preoperative) clinical characteristics of the study patients

	Circumferential group *N* = 82 eyes	Segmental group *N* = 84 eyes	*P* value
Age (mean ± standard deviation) (years)	56.5 ± 5.0	55.9 ± 5.2	0.526
Male / female	47/35	51/33	0.512
Eyes (Unilateral)	82 (23)	84 (25)	
Unilateral (right/left)	13/10	12/13	
Bilateral	59 (36 males)		
BCVA (mean ± standard deviation)	0.50 ± 0.1	0.49 ± 0.1	0.409
IOP (mmHg)	32.3 ± 2.4	31.8 ± 2.6	0.234
AGM (mean ± standard deviation)	3.2 ± 0.6	3.2 ± 0.6	0.468
cup disc ratio (mean ± standard deviation)	0.8 ± 0.1	0.8 ± 0.1	0.926
Mean Deviation (mean ± standard deviation) (dB)	-11.3 ± 5.4	-10.9 ± 6.3	0.639
Pattern Standard Deviation (mean ± standard deviation) (dB)	10.5 ± 4.8	10.5 ± 4.8	0.979
RNFL thickness (mean ± standard deviation) (µ)	72.7 ± 12.7	71.7 ± 12.0	0.618
Follow up period (mean ± standard deviation) (months)	38.4 ± 8.3	38.5 ± 8.0	0.912

2 Randomized Controlled Trial of Circumferential Versus Segmental Ab externo Angle Surgery in Patients with Primary Open Angle Glaucoma

*BCVA*  best corrected visual acuity, *IOP*  intraocular pressure, *AGM * anti-glaucoma medications, *RNFL*  retinal nerve fiber layer. (Test used = unpaired t-test. Significant at *p* < 0.05)

**Table 2:** Postoperative clinical parameters of the study groups

	Circumferential group (*N* = 82 eyes)	Segmental group (*N* = 84 eyes)	*P* value
Preoperative IOP (mean ± standard deviation) (mmHg)	32.3 ± 2.4	31.8 ± 2.6	0.234
Postoperative IOP (mean ± standard deviation) (mmHg) (n = number of eyes available at follow up)			
1st week	10.8 ± 1.1 (*n* = 82)	10.6 ± 1.1 (*n* = 84)	0.211
1st month	11.8 ± 0.9 (*n* = 82)	11.4 ± 0.9 (*n* = 84)	0.004
2nd month	12.2 ± 0.9 (*n* = 79)	12.0 ± 0.9 (*n* = 81)	0.081
3rd month	12.4 ± 1.0 (*n* = 79)	13.0 ± 1.0 (*n* = 81)	0.001
6th month	12.5 ± 1.3 (*n* = 79)	13.3 ± 1.1 (*n* = 81)	< 0.001
9th month	12.8 ± 1.8 (*n* = 79)	13.9 ± 1.5 (*n* = 81)	< 0.001
12th month	12.8 ± 2.1 (*n* = 78)	14.1 ± 1.6 (*n* = 81)	< 0.001
15th month	12.8 ± 2.1 (*n* = 78)	14.4 ± 2.2 (*n* = 81)	< 0.001
18th month	12.5 ± 1.1 (*n* = 75)	14.6 ± 2.2 (*n* = 77)	< 0.001
21st month	12.4 ± 1.0 (*n* = 75)	14.8 ± 1.6 (*n* = 76)	< 0.001
24th month	12.4 ± 1.2 (*n* = 75)	15.5 ± 0.9 (*n* = 74)	< 0.001
Reduction in IOP (mmHg)	19.9 ± 2.6	16.3 ± 2.6	< 0.001
% of reduction of IOP (mmHg)	61.5 ± 4.3	51.2 ± 4.8	< 0.001
Postoperative AGM	0.5 ± 1.2	0.7 ± 1.3	0.346
BCVA (decimal)	0.54 ± 0.1	0.52 ± 0.1	0.184
VF-MD (dB)	-11.15 ± 5.3	-11.30 ± 5.5	0.861
VF-PSD (dB)	10.57 ± 4.9	10.7 ± 4.9	0.886

*IOP *  intraocular pressure, *AGM*  anti-glaucoma medications. *VF-MD* visual field mean deviation, *VF-PSD* visual field pattern standard deviation

Test used = unpaired t-test. Significant at *p* < 0.05

**Fig. 2:**
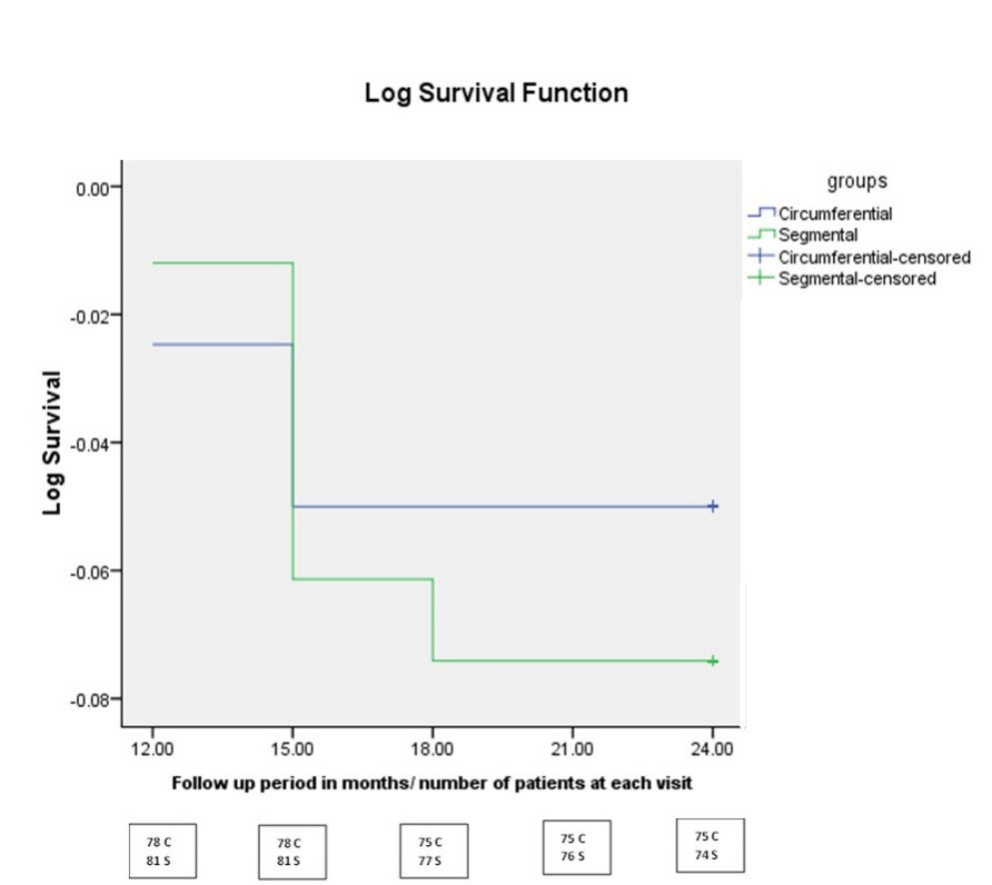
The distributions of the baseline tomographic and biomechanical parameters for both groups (Non-progressed and Progressed)

The Kaplan-Meier survival curve demonstrates that the complete surgical success rates at 12, 15, 18 and 24 months were 98.8%, 96.4%, 95.1% and 85.4% in the circumferential group and 98.8%, 92.9%, 91.7%, 79.8% in the segmental group respectively. There was no statistically significant difference between the 2 groups regarding the complete success rate at 24 months postoperatively (log-rank test, *p* = 0.331) (Fig. 2). Additionally, based on the definition of success of IOP between 6 and 14 mmHg and with at least 40% reduction from baseline IOP, the success percentage at 24 months was 93.9% (8.5% qualified) in the circumferential group and 23.8% (9.5% qualified) in the segmental group. The complications encountered in both study groups are presented in Table 3. A minimal self-limiting hyphema was a universal occurrence in all study eyes. This occurred in 55 eyes (67.1%) in the circumferential group and in 63 eyes (75.0%) in the segmental group (grade I) and resolved in less than 7 days. A grade II hyphema (involving 1/3 to ½ of the AC) occurred in 23 eyes (28.0%) in the circumferential group and in 19 eyes (22.62%) in the segmental group and resolved spontaneously in 9–12 days. A grade III hyphema (involving more than ½ of the AC) occurred in 4 eyes (4.9%) in the circumferential group and in 2 (2.38%) in the segmental group and these necessitated AC wash (*p* = 0.457). A localized stripping of the Descemet membrane (8 eyes) and transient IOP spikes (8 eyes) were the most common complications encountered in the segmental group, whereas transient hypotony (IOP < 6 mmHg) (6 eyes) was the most common complication in the circumferential group. Subsequent surgical procedures are presented in Table 4 which demonstrates that 7 eyes in the segmental group needed a subsequent inferior trabeculotomy. To further refine the results of the comparison of the circumferential group to the segmental group, the subset of patients in whom both eyes were included in the study (59 patients with one eye randomized to either procedure and the other eye subjected to the other procedure), were analyzed separately and the results presented in Table 5 (clinical characteristics at the preoperative and final postoperative visits) and Table 6 (IOP changes at different follow up time points). There were no statistically significant differences between the 2 groups except in the IOP measurements over time from the first month onwards, with the circumferential group presenting marginally higher IOP values up to the 2nd postoperative month followed by a consistent reduction than the segmental group from the 3rd postoperative month onwards.

**Table 3:** Complications encountered in study eyes

			Circumferential group (*N* = 82 eyes %)	Segmental group (*N* = 84 eyes %)	*P* value
Hyphema			82(100)	84 (100)	1.0
Grade of Hyphema	**Level of hyphema**	**Time of resolution of hyphema**			
Grade I	**<** 1/3 of AC	< 7 days	55 (67.1)	63 (75.0)	
Grade II	From 1/3 to ½ of AC	9–12 days	23 (28.0)	19 (22.62)	
Grade III	**>** 1/2 of AC	Removed by I/A	4 (4.9)	2 (2.38)	
Descemet’s membrane split			0 (0)	8 (9.5)	**0.007**
Shallow anterior chamber			1 (1.22)	2 (2.38)	1.0
Self-limited ciliochoroidal Detachment			1 (1.22)	2 (2.38)	1.0
Transient hypotony (IOP < 6 mmHg)			6 (7.32)	5 (5.95)	0.765
Transient IOP spike (IOP > 24 mmHg)			2 (2.44)	8 (9.5)	0.099
Bleb formation			1 (1.22)	4 (4.76)	0.368
Progression of cataract			3 (3.66)	5 (5.95)	0.720

**Table 4:** Additional surgeries in the study eyes

	Circumferential group (*N* = 82 eyes)	Segmental group (*N* = 84 eyes)	P value
Inferior trabeculotomy	0	7	0.535
Trabeculectomy	4	0	
Goniosynechiolysis	0	2	
Transscleral diode cyclophotocoagulation	2	2	
Total of glaucoma procedures	**6 (7.3%)**	**11 (13.1%)**	
Anterior chamber irrigation	3	2	
Phacoemulsification	2	3	
Total	**11 (19.5%)**	**16 (19.0%)**	

**Table 5:** Between eyes comparison in patients with bilateral surgery (clinical characteristics)

(mean ± Standard Deviation)	Circumferential Group (*N* = 59)	Segmental Group (*N* = 59)	*P* value
Pre-operative CD	0.75 ± 0.1	0.77 ± 0.1	0.354
Post-operative CD	0.74 ± 0.1	0.76 ± 0.1	0.305
Pre-operative RNFL (u)	73.58 ± 13.1	71.02 ± 12.37	0.277
Post-operative RNFL (u)	75.813.1	72.1 ± 12.1	0.115
Pre-operative BCVA (decimal)	0.5 ± 0.1	0.5 ± 0.1	0.858
Post-operative BCVA (decimal)	0.5 ± 0.1	0.5 ± 0.1	0.335
Pre-operative MD (dB)	-10.89 ± 5.1	-10.83 ± 6.7	0.964
Post-operative MD (dB)	-10.7 ± 4.8	-11.4 ± 5.3	0.409
Pre-operative PSD (dB)	10.11 ± 4.5	10.8 ± 4.8	0.460
Post-operative PSD (dB)	10.1 ± 4.3	10.8 ± 4.6	0.359
Pre-operative IOP (mmHg)	32.4 ± 2.3	31.9 ± 2.5	0.345
IOP reduction (mmHg)	19.9 ± 2.4	16.3 ± 2.6	< 0.001
% reduction in IOP (mmHg)	61.3 ± 4.1	51.2 ± 5.0	< 0.001
Pre-operative AGM	3.3 ± 0.6	3.2 ± 0.6	0.642
Post-operative AGM	0.6 ± 1.3	0.8 ± 1.4	0.500

*CD* cup/disc ratio, *RNFL* retinal nerve fiber layer, *BCVA* Best corrected visual acuity, *IOP*  intraocular pressure, *AGM*  anti-glaucoma medications. *MD:* mean deviation, *PSD* pattern standard deviation

Test used = unpaired t-test. Significant at *p* < 0.05

**Table 6:** Between eyes comparison in patients with bilateral surgery (IOP over follow up time points)

(mean ± Standard Deviation)	Circumferential Group (*N* = 59)	Segmental Group (*N* = 59)	*P* value
1st week	10.9 ± 1.0	10.6 ± 1.1	0.131
1st month	11.9 ± 0.9	11.3 ± 0.9	0.001
2nd month	12.4 ± 0.9	12.0 ± 0.8	0.009
3rd month	12.6 ± 1.0	13.0 ± 1.0	0.017
6th month	12.7 ± 1.3	13.4 ± 1.1	0.009
9th month	13.0 ± 2.0	14.0 ± 1.6	0.002
12th month	13.1 ± 2.3	14.3 ± 1.7	0.003
15th month	13.0 ± 2.4	14.6 ± 2.4	0.001
18th month	12.7 ± 1.2	14.6 ± 2.2	< 0.001
21st month	12.7 ± 1.2	14.9 ± 1.8	< 0.001
24th month	12.8 ± 1.5	15.4 ± 1.1	< 0.001

## Discussion

The current study compared VCST to segmental VT in the surgical treatment of POAG. Both procedures were effective in lowering the IOP for up to 2 years after the surgery, although VCST provided more reduction of IOP till the last follow up. Studying the demographic characteristics of the study patients revealed a slight male predominance in both study groups, paralleling other published studies [2, 21, 22] citing POAG to be more prevalent in men than women. Both the sex distribution and the age groups were almost identical in both study groups, rendering the results comparable and the findings more robust and reliable. Likewise is the similarity between the study groups in the preoperative clinical characteristics, the BCVA, IOP, AGM, C/D ratio, VF global indices and the pRNFL. The indication for surgery in the present study was an uncontrolled IOP despite maximally tolerated AGM, the most commonly reported indication for surgery in POAG [23]. Additional evidence of the advanced stage of POAG is the advanced C/D ratio and the VF global indices (MD and PSD) and this justifies the authors’ attempts to aim for a low target IOP to control the advanced stages of the disease. The fact that the follow up extended for 2 years rendered the study findings robust, especially given the low dropout rate in the study cases. Following the IOP trend in the study eyes demonstrates clearly the marked efficacy of both procedures in lowering the IOP and for a sustained duration of at least 2 years, though the circumferential group demonstrated a trend towards lower IOP than the segmental group from the 6th postoperative month onwards, paralleling other published reports [24] that the more circumference of the angle incised, the lower the IOP. The same findings are emphasized by comparing the 2 procedures in both eyes of the same patients. On another note, 7 eyes in the segmental group required the incision of an additional circumference of the angle (inferior trabeculotomy) to bring about IOP control emphasizing the beneficial effect of increasing the extent of the incised angle to bring about further lowering of the IOP. The relative superiority of the circumferential procedure over the segmental procedure is further inferred by the finding that almost twice the number of eyes in the segmental group needed a subsequent glaucoma procedure (13%) than in the circumferential group (7%). This finding of the superiority of IOP control with increasing the circumference of the treated angle contrasts with the study by Zhang et al. [25] reporting on the influence of goniotomy size on the IOP in eyes with POAG. Contrary to the current study findings, Zhang et al. report no increased benefit on IOP control with increased angle circumference treated but only increased incidence of hyphema. These apparently different outcomes highlight the need for more randomized controlled trials in this relatively novel approach to POAG treatment.

The beneficial effect of IOP lowering on the VF and visual function encountered in both study groups parallels other reports [26].

Revision of the complications encountered in the study eyes reveals important insights. Hyphema is a universal occurrence in angle procedures, as already reported in previous studies [17], a backflow of blood from the episcleral plexus consequent to reduction of IOP. An interesting finding is the localized Descemet membrane split encountered in the segmental group, an occurrence reported by the authors in a previous publication [13]. The fact that it did not occur in the circumferential group is rather intriguing and may be related to the direction of the sweep through Schlemm’s canal into the anterior chamber. Although the procedures studied were not filtering procedures, inadvertent blebs were encountered in a minority of the operated eyes, notably more in the segmental group, though not statistically significant. The information inferred by the study findings can be construed as complete only by the subsequent procedures performed on the study eyes. The importance of increasing the extent of the circumference of the incised (treated) angle is exemplified by the fact that 7 eyes in the segmental group needed an additional angle procedure. Finally, studying the success of both procedures reveals that both were successful in IOP control, though the circumferential procedure provided significantly greater reduction of IOP, with a greater longevity of the procedure, in accordance with the published reports of the greater efficacy of circumferential procedures over segmental procedures [24, 27].

A significant strength in the current study is the presence of a cohort of patients in which one eye was subjected to one procedure and the other subjected to the other, allowing comparison of both procedures in the same patients. The findings in this specific cohort of patients echoes the findings in the whole study cohort in which both procedures were effective -with the circumferential procedure more effective than the segmental in bringing about more IOP reduction throughout the follow up period.

This study is not without limitations. The glaucoma diagnosis being essentially limited to POAG does not allow generalization of the findings to other glaucoma types.

In conclusion, angle procedures -segmental and circumferential- are effective in lowering the IOP for at least 2 years in eyes with POAG, with circumferential angle surgery providing greater reduction of IOP and requiring less subsequent glaucoma procedures.
